# Therapeutic Landscape of HPV-Associated Cancers: From Mechanisms and Conventional Approaches to Future Innovations

**DOI:** 10.3390/cancers18040636

**Published:** 2026-02-15

**Authors:** Muneera Anwer, Krupa Bhaliya, Memoona Zahra, Urooj Yousaf Virk, Hafiza Aasia Malik, Ming Q. Wei

**Affiliations:** School of Pharmacy and Medical Science, Griffith University, Gold Coast Campus, Gold Coast, QLD 4215, Australia

**Keywords:** human papillomavirus, HPV-associated cancers, viral oncoproteins, E6, E7, immunotherapy, vaccines

## Abstract

Cancer remains the second leading cause of mortality worldwide. Human papillomavirus is a widespread DNA virus with well-established oncogenic potential, particularly among high-risk genotypes such as HPV16 and HPV18. Persistent infection with these genotypes can lead to the development of several malignancies, including cervical, oropharyngeal, anal, penile, vulvar, and vaginal cancers. Cervical cancer persists as the most prevalent malignancy associated with HPV infection, disproportionately affecting low- and middle-income countries. This review provides an overview of the HPV genome, viral genotypes, and associated malignancies, with particular emphasis on the viral oncoproteins E6 and E7 and the mechanisms of viral DNA integration into the host genome. In addition, recent advances in diagnostic technologies, therapeutic strategies, ongoing clinical trials, and future directions to reduce the global burden of HPV-related cancers are discussed.

## Simple Summary

Human papillomavirus (HPV) is a common viral infection that can lead to several types of cancer, including cervical, anal, vulvar, vaginal, and certain head and neck cancers. Although preventive vaccines have significantly reduced new infections, many HPV-related cancers still occur worldwide. This review explains how HPV causes cancer, outlines the different cancer types linked to the virus, and discusses current strategies for prevention, screening, and treatment. We also highlight recent advances in molecular diagnostics and emerging therapies, including immune-based treatments and therapeutic vaccines. By bringing together current knowledge on prevention and treatment, this article aims to clarify the evolving clinical landscape of HPV-associated cancers and identify areas for further research. A better understanding of these developments may support improved patient outcomes and guide future innovation in cancer therapy.

## 1. Human Papillomavirus

**Figure 1:**
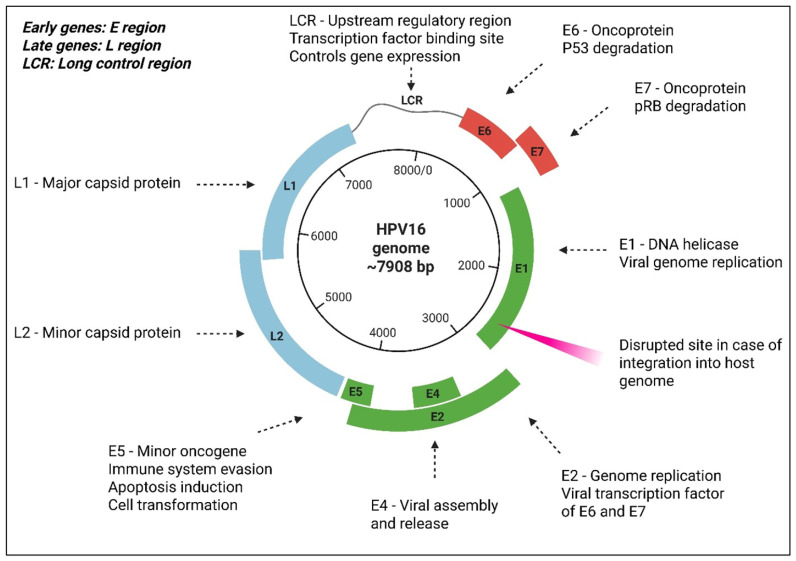
Genomic structure of HPV16. Dashed arrows represent early genes (green), late genes (blue), oncoproteins (red), and a long control region, which together regulate viral replication and expression.

Human papillomavirus (HPV) belongs to the family *Papillomaviridae* and comprises small, non-enveloped double-stranded DNA viruses. Papillomaviruses possess a genome of approximately 8000 base pairs. HPV can infect squamous epithelium, or cells with the potential for squamous maturation, leading to the development of proliferative lesions. These viruses exhibit species specificity and strong tissue tropism, primarily targeting the skin surface or internal squamous mucosal tissues [1]. Until now, researchers have identified approximately 130 or more types of HPV, primarily through sequencing the genes encoding the capsid protein L1 [2]. The HPV genome is organised into three main regions: a noncoding upstream regulatory region (URR) of approximately 1 kilobase, an early region containing open reading frames (ORFs) E1, E2, E4, E5, E6, and E7, and a late region that encodes the L1 and L2 proteins, which form components of the viral capsid [3]. Figure 1 illustrates the genomic organisation of the HPV virus. Among these proteins, E1, E2, E4, and E5 are essential for viral DNA replication. The oncogenic proteins E6 and E7 work together to transform infected cells, contributing to cancer development. Meanwhile, L1 and L2 proteins are crucial for the assembly of HPV viral particles and the formation of virus-like particles (VLPs) [4,5]. VLPs are large structures composed of one or more structural proteins from various viruses. They lack viral nucleic acids and are therefore incapable of independent replication. Despite this, they closely resemble actual viral particles in overall structure.

HPV contributes significantly to the development of multiple types of cancer. It has been linked to both benign and malignant neoplasms. HPV comprises nearly 200 related viruses; they are categorized as high-risk and low-risk genotypes in correlation with HPV-associated lesions that undergo malignant transformation. High-risk HPV genotypes such as HPV 16 and 18 are strongly associated with several human cancers [6]. Low-risk HPV types, such as HPV6 and HPV11, are rarely oncogenic but are commonly associated with benign lesions, including anogenital and oropharyngeal warts [7]. This review highlights current knowledge on human papillomavirus, including its classification and the spectrum of associated malignancies. The focus is on the molecular mechanisms underlying HPV-induced carcinogenesis, notably the roles of the viral oncoproteins E6 and E7, as well as the integration of HPV DNA into the genome of the host. Furthermore, this review underscores recent advances in diagnostic methodologies and therapeutic strategies and highlights ongoing clinical trials. By establishing emerging research and future directions, this work aims to support the development of more effective interventions for reducing the global burden of HPV-related cancers.

## 2. Modes of HPV Transmission

HPV is predominantly transmitted through sexual contact, including vaginal, oral, and anal intercourse. Nonetheless, non-sexual routes such as vertical transmission during childbirth and skin-to-skin contact have also been implicated in the spread of the virus [8]. Following transmission, HPV targets the basal epithelial cells, where it can establish a persistent infection. In most cases, these infections are transient and resolve spontaneously by the host’s immune system in 1 to 2 years, typically without clinical manifestation or long-term consequences [9]. However, persistent infection with high-risk HPV genotypes could induce dysplastic transformations in the epithelium, potentially progressing to high-grade lesions and, ultimately, malignant transformation if left untreated [10].

## 3. HPV–Host Interaction

**Figure 2:**
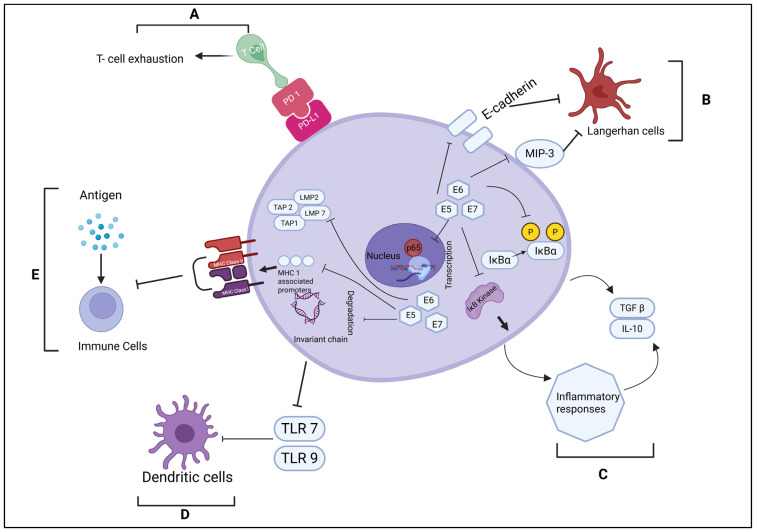
Mechanisms of Immune Evasion by HPV-Related Tumour Cells. (**A**) HPV-associated tumour cells express PD-L1, which binds to PD-1 receptors on T cells, leading to T cell exhaustion and immune suppression. (**B**) HPV affects both the number and activity of Langerhans cells (LCs) by lowering E-cadherin expression and interfering with MIP-3 transcription, which are essential for LC function and recruitment. (**C**) HPV inhibits the activation of IκB kinase and the phosphorylation of IκBα, disrupting NF-κB p65-mediated transcription. This manipulation of the NF-κB pathway reduces effective inflammatory responses and promotes the release of immunosuppressive cytokines such as IL-10 and TGF-β. (**D**) HPV downregulates Toll-like receptors TLR7 and TLR9, preventing the activation of dendritic cells (DCs). Consequently, HPV remains undetected by the immune system. (**E**) The viral proteins E5, E6, and E7 reduce the expression of MHC class I and II molecules via repressing MHC class I-associated promoters, blocking the degradation of the invariant chain, and inhibiting components of the antigen processing machinery, ultimately impairing antigen presentation. Solid forward arrows indicate activation or progression of a biological process. Blocking (blunt-ended) arrows represent inhibition, downregulation, or reduced expression of the indicated target.

Human papillomavirus enters the host through disruptions or micro abrasions in the epithelial barrier of the cells. The initial attachment involves binding to cell surface receptors, including heparan sulphate proteoglycans and more specific receptors such as α6-integrin, which facilitate subsequent internalisation of the viral particle [11]. Once internalised, HPV employs a range of immune evasion strategies to persist within the host. These include suppression of interferon-mediated signalling pathways, interfering with antigen presentation and processing, and modulating the functions of adaptive and innate immune cells [12]. Through these mechanisms, the virus circumvents immune clearance as shown in Figure 2, thereby establishing persistent infections that are strongly associated with disease progression [13]. Following entry, HPV utilises the host cellular machinery for the transcription and replication of its genome. Viral gene expression is tightly regulated temporally to ensure stage-specific production of viral proteins required for replication and persistence [11]. Among high-risk HPV genotypes, notably HPV16 and HPV18, the viral oncoproteins E6 and E7 are central to malignant transformation [12,14]. This dysregulation of host cell-cycle checkpoints by viral oncoproteins fosters unchecked cellular proliferation and genomic instability, both critical steps in oncogenesis. HPV replication is intricately synchronised with the host cell cycle, particularly during the S phase, when the host DNA replication machinery is active. The virus exploits host enzymes and regulatory factors to ensure efficient duplication of its genome, maintaining coordination with host DNA synthesis [8]. Importantly, HPV replication is closely linked to the differentiation state of epithelial cells. As infected basal cells differentiate and migrate toward the superficial epithelial layers, a shift in viral gene expression leads to the synthesis of late genes encoding structural proteins, culminating in the assembly of progeny virions [11]. The host immune response plays a pivotal role in controlling HPV infection. Both innate and adaptive immune mechanisms are involved in recognising and eliminating infected cells. Cytotoxic T lymphocytes, natural killer cells, and professional antigen-presenting cells contribute to viral clearance through recognition of infected epithelia and the orchestration of targeted immune responses [15,16].

## 4. Role of Viral Oncoprotein in Carcinogenesis

The primary critical event during persistent infection with high-risk HPV genotypes is the integration of the viral genome into the host cellular DNA [17]. HPV E5 oncoprotein contributes to early stages of cellular transformation by modulating growth factor receptor signalling and enhancing cell proliferation. One of its key oncogenic functions is the potentiation of epidermal growth factor receptor (EGFR) signalling through inhibition of receptor degradation and enhanced receptor recycling, resulting in prolonged mitogenic signalling. E5 has also been shown to interfere with acidification of endosomes by interacting with the vacuolar H^+^-ATPase, thereby sustaining growth factor receptor activation. Through these mechanisms, E5 promotes cell cycle progression, cooperates with E6 and E7 to enhance proliferative signalling, and contributes to the early phases of HPV-mediated carcinogenesis before viral genome integration. Although E5 expression is frequently lost following viral integration in advanced cancers, its role in establishing a pro-proliferative cellular environment is considered critical during early infection and lesion development [18,19,20,21].

**Figure 3:**
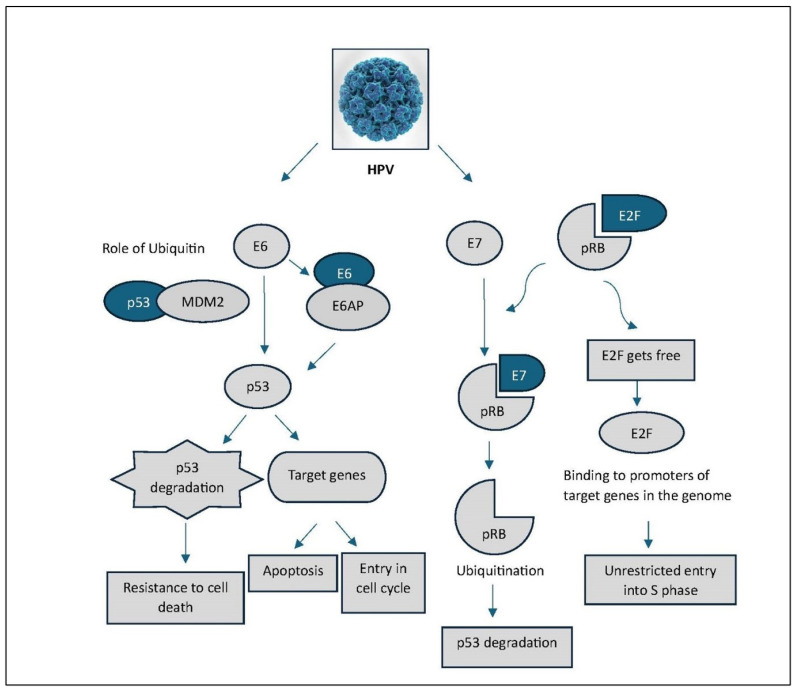
Mechanism of HPV-mediated disruption of cell cycle regulation through degradation of tumour suppressors p53 and pRb.

The E6 oncoprotein of high-risk HPV types targets the tumour suppressor protein p53 for ubiquitin-mediated degradation. Given p53’s essential role in regulating the cell cycle, facilitating DNA repair, and inducing apoptosis in response to genomic stress, its functional inactivation permits the survival and propagation of cells harbouring DNA damage [13,22]. Concurrently, the E7 protein binds to and functionally inactivates the retinoblastoma (Rb) protein, a critical gatekeeper of the G1/S cell cycle checkpoint. Rb normally inhibits cell cycle progression by sequestering E2F transcription factors; E7-mediated Rb inactivation results in uncontrolled entry into S phase and aberrant cellular proliferation [23]. Figure 3 illustrates the mechanism of HPV-mediated disruption of cell cycle regulation through degradation of tumour suppressors p53 and pRb. The figure depicts how high-risk HPV oncoproteins E6 and E7 promote oncogenesis by targeting key tumour suppressors. E6 forms a complex with E6AP, leading to the ubiquitin-mediated degradation of p53, either directly or through modulation of the MDM2 pathway, thereby inhibiting apoptosis and promoting resistance to cell death. E7 binds to the retinoblastoma protein (pRb), resulting in its ubiquitination and degradation. This releases E2F transcription factors, allowing them to drive transcription of genes required for S-phase entry, thereby promoting uncontrolled cell cycle progression. The coordinated inactivation of p53 and pRb pathways by HPV enables evasion of cell-cycle checkpoints and contributes to cellular transformation. Together, the oncogenic activities of E6 and E7 promote cellular transformation, characterised by sustained proliferative signalling, evasion of growth suppressors, and the loss of normal differentiation programs. These transformed cells progressively accumulate genetic and epigenetic alterations that drive malignant progression [24,25]. Beyond p53 and Rb, HPV oncoproteins interact with a range of host cellular factors, perturbing critical processes such as DNA damage response, apoptosis regulation, cell adhesion, and immune signalling [15,26]. Although HPV infection typically triggers an immune response involving the recruitment of immune effector cells to the site of infection, high-risk HPV types have evolved sophisticated mechanisms to avoid immune surveillance. These include the downregulation of antigen presentation pathways and the modulation of cytokine signalling. As a result, persistent infection is maintained, often accompanied by chronic inflammation, which further contributes to tumour initiation and progression through enhanced cell survival and proliferative signalling [9,16]. If left untreated, HPV-associated premalignant lesions such as cervical intraepithelial neoplasia (CIN) may progress to invasive carcinoma. The transition from precancerous to malignant states is driven by the gradual accumulation of additional genomic alterations, including inactivation of tumour suppressor genes and activation of oncogenes, culminating in the development of invasive cancer [14].

## 5. Integration of Viral DNA into the Host Genome

**Figure 4:**
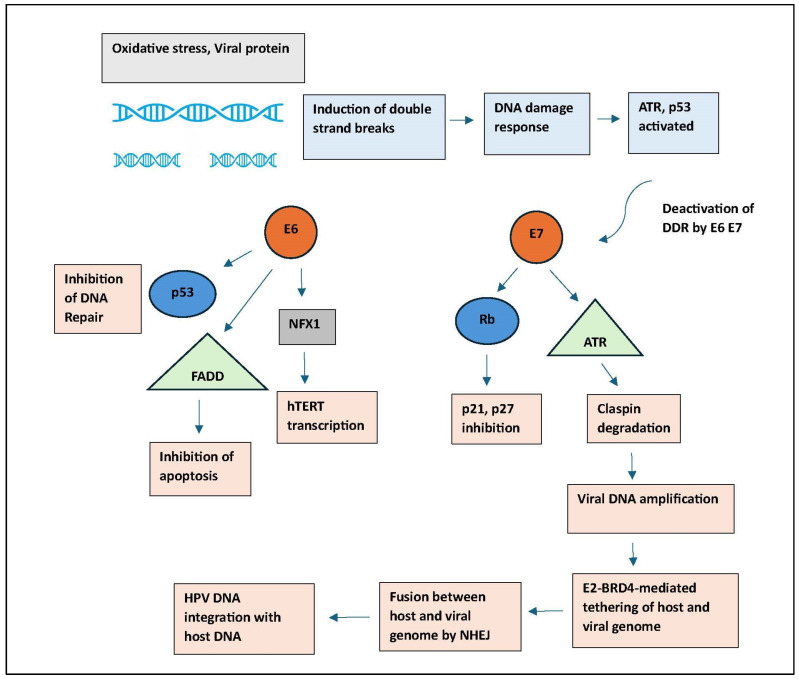
Mechanism of HPV-Induced Genomic Instability and Viral DNA Integration. The blue arrow indicates double-stranded breaks.

The integration of HPV DNA into the host genome is a pivotal event in the progression from infection to malignancy. This process often disrupts the viral E1 and E2 genes, which normally regulate the expression of the viral oncogenes E6 and E7 [27]. Loss of E2 leads to uncontrolled expression of E6 and E7 [28]. Importantly, multiple HPV DNA copies may integrate into the genome; typically, only one integration site is transcriptionally active, giving rise to virus–host fusion transcripts. These transcripts use host splicing machinery and a host-derived polyadenylation signal downstream of the integration point to ensure stable expression of E6 and E7 [29]. The precise location and genomic context of the integration site, such as proximity to active chromatin, enhancers, or regulatory elements, significantly influence the expression of the viral oncogenes [30]. The active integration site becomes a focal point for clonal expansion, as cells harbouring this single active integration gain a proliferative advantage. This highlights that HPV integration is not merely a passive event but rather a functional driver of carcinogenesis, making it a potential target for therapeutic intervention in HPV-related cancers. A study highlights that, despite the existence of multiple integrated HPV DNA copies, only one integration site is transcriptionally active, producing virus–host fusion transcripts [29]. These fusion transcripts utilize host cellular machinery, including splicing and polyadenylation signals, to ensure the stability and translation of E6 and E7 mRNAs. The specific genomic context of the integration site influences the efficiency of oncogene expression, suggesting that the surrounding host DNA elements contribute to modulating viral gene expression [31]. This finding underscores the significance of virus–host fusion transcripts in disrupting host cell regulation and promoting oncogenic processes [32,33]. Figure 4 is a schematic representation of the molecular events following HPV infection, leading to genomic instability and integration of viral DNA into the host genome. Oxidative stress and viral proteins induce DNA double-strand breaks (DSBs), triggering a DNA damage response (DDR) involving ATR and p53 activation. The HPV oncoproteins E6 and E7 interfere with DDR by degrading or inactivating key tumour suppressors: E6 promotes degradation of p53 and inhibition of apoptosis via FADD, while also enhancing hTERT transcription through NFX1. E7 inactivates Rb, inhibits p21/p27, and promotes Claspin degradation, suppressing ATR function and facilitating viral DNA amplification. Persistent infection and DDR suppression lead to the integration of HPV DNA into the host genome via non-homologous end joining (NHEJ), resulting in host-viral genome fusion. E2-BRD4-mediated tethering stabilizes this fusion, contributing to oncogenic transformation.

## 6. Development of Malignancy

The persistent overexpression of HPV oncogenes E6 and E7, driven by a single active integration site, is a pivotal factor in the development of malignancies [34]. Silencing the virus–host fusion transcripts at this active site results in the cessation of cell growth and the induction of senescence, highlighting the critical role of this integration event in maintaining the malignant phenotype [29]. These findings suggest that targeting the specific virus–host fusion transcripts could be a viable therapeutic strategy, offering a more precise approach to treating HPV-associated cancers by disrupting the essential oncogenic drivers at their source.

## 7. HPV and Cancer

A unique pattern is observed in the correlation between cancer burden and human papillomavirus, with economic development as a key factor. The Age-Standardized Incidence Rate (ASIR) for these cancers varies from 6.9 cases per 100,000 person-years in high-income countries to 16.1 in low-income countries [35]. High risk-HPV types 16, 18, 31, 33, 34, 35, 39, 45, 51, 52, 56, 58, 59, 66, 68, and 70 represent the strains most frequently associated with cancer development, while low risk-HPV 6, 11, 40, 42, 43, 44, 55, 61, 81, and 83 are associated with genital warts. Across diverse regions and among individuals of varying sexual orientations, high-risk HPV is linked to a significant proportion of various malignancies [17].

### 7.1. Smoking as a Co-Factor in HPV-Mediated Carcinogenesis

Tobacco smoking acts as an important environmental cofactor that modifies the natural history of HPV infection and enhances carcinogenic risk in HPV-associated malignancies. Population surveillance data demonstrate that the incidence of HPV-positive cervical, anal, and oropharyngeal cancers is significantly higher among individuals who have ever smoked compared with never-smokers, supporting a modifying role of tobacco exposure in HPV carcinogenesis [36]. Smoking has been associated with increased persistence of high-risk HPV infections, likely mediated through impaired immune responses that reduce HPV clearance, and is independently linked with increased risk of histopathological abnormalities in HPV-positive cervical tissues [37]. In high-risk HPV-positive women, prolonged and excessive smoking significantly exacerbates HPV persistence and progression to neoplastic changes, suggesting that tobacco exposure amplifies viral oncogenic activity [38]. Genetic evidence from recent multivariable Mendelian randomization analyses indicates that lifetime smoking exposure increases the risk of both HPV-positive and HPV-negative head and neck cancer [39]. Additionally, systematic reviews show that smoking is associated with worse clinical outcomes in HPV-positive oropharyngeal cancer, including reduced survival and increased recurrence, effectively diminishing the favourable prognosis typically conferred by HPV positivity [40]. Mechanistically, components of tobacco smoke may promote genomic instability, chronic inflammation, and immune suppression, thereby facilitating HPV persistence and malignant progression in epithelial tissues. Although the precise molecular mechanisms remain under continued investigation, the convergence of observational, genetic, and clinical outcome data underscores the significant role of smoking as a modifying risk factor in HPV-mediated carcinogenesis across multiple anatomic sites.

### 7.2. Anogenital Cancers

#### 7.2.1. HPV and Cervical Cancer

**Figure 5:**
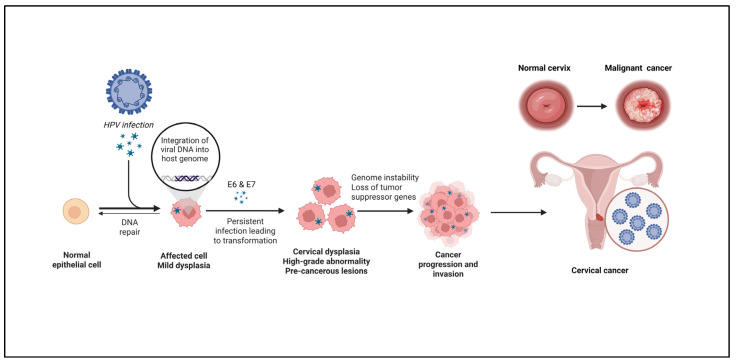
HPV infection leading to cervical malignancy. Persistent HPV infection leads to viral DNA integration into the host genome, resulting in cervical cancer.

According to the International Agency for Research on Cancer (IARC), cervical cancer is one of the most prevalent malignancies associated with oncogenic strains of HPV, accounting for 95% of cervical cancer cases. Cervical cancer primarily comprises squamous cell carcinoma (SCC) and a smaller proportion of adenocarcinomas [41]. High-risk HPV types 16 and 18 account for most cases, with HPV16 alone responsible for over 60% of cervical cancers worldwide [42]. In 2020, an estimated 604,000 new cases of cervical cancer were diagnosed globally, resulting in approximately 342,000 deaths [43]. Cervical cancer is the fourth most common cancer among women, with nearly 90% of deaths occurring in low- and middle-income countries (LMICs), where access to organized screening and HPV vaccination remains limited. The disease disproportionately affects women under 50 years of age, reflecting gaps in prevention and early detection strategies [44,45]. In response, the World Health Organization (WHO) has launched a global initiative to eliminate cervical cancer through expanded HPV vaccination, improved screening coverage, and timely treatment of precancerous lesions [46]. HPV infection is highly prevalent, with approximately 50–80% of sexually active individuals acquiring at least one genital HPV infection during their lifetime. However, less than 1% of infections progress to malignancy, as most are transient and resolve spontaneously [47]. Persistent infection with high-risk HPV—defined as detection of the same HPV DNA in samples collected 6–12 months apart—is a key prerequisite for cervical carcinogenesis [48]. Women living with HIV are at a six-fold increased risk of developing cervical cancer due to impaired immune clearance of HPV [49]. HPV infects cervical epithelial cells, inducing cellular and molecular alterations that may progress to cervical intraepithelial neoplasia and, if untreated, invasive cancer, as illustrated in Figure 5. Cervical cancer development is influenced by a complex interplay of viral, host, and environmental factors, including early sexual debut, multiple sexual partners, prolonged hormonal contraceptive use, smoking, co-infection with sexually transmitted pathogens, low socioeconomic status, and inadequate antioxidant intake [50]. Although host immune and genetic factors, as well as viral characteristics, are known to contribute to disease initiation and progression, their precise roles are still unidentified [51].

#### 7.2.2. HPV and Anal Cancer

The association between HPV infection and anal cancer is well established. Although anal cancer remains relatively uncommon, its incidence has increased in recent decades, particularly among high-risk populations. Men who have sex with men and individuals living with HIV exhibit a significantly higher risk of developing anal malignancies compared with the general population. Anal intraepithelial neoplasia (AIN) is widely regarded as a precursor lesion to anal cancer; however, the natural history of AIN progression remains less clearly defined than that of cervical intraepithelial neoplasia. Most available evidence is derived from small cohort studies with limited follow-up periods (5–10 years) [52]. Consequently, larger longitudinal studies are required to better characterize the progression of AIN to invasive anal cancer and to determine its implications for disease management and treatment outcomes.

#### 7.2.3. HPV and Vulvovaginal Cancer

Like other HPV-associated malignancies, vulvar and vaginal cancers occur more frequently in younger women and share pathogenic mechanisms with cervical cancer. High-risk HPV infection plays a central role in the development of HPV-associated vulvar squamous cell carcinoma (SCC). A systematic review reported a 3.3% progression rate from vulvar intraepithelial neoplasia (VIN) to invasive vulvar SCC [53]. Evidence from large clinical trials further supports the role of HPV in vulvovaginal malignancies. The Females United to Unilaterally Reduce Endo/Ectocervical Disease-(FUTURE) I and II studies demonstrated a substantial reduction in HPV-related anogenital infections among young women following prophylactic HPV vaccination, underscoring the preventive potential of vaccination against HPV-associated vulvovaginal cancers [54].

#### 7.2.4. HPV and Head and Neck Cancers

The incidence of HPV-associated head and neck squamous cell carcinoma (HNSCC) has increased markedly over recent decades, particularly among younger individuals [55,56]. HNSCC is the sixth most common malignancy worldwide and most frequently arises in the oral cavity, oropharynx, nasopharynx, larynx, and hypopharynx [57]. The disease shows a strong male predominance, with incidence rates approximately two- to three-fold higher in men than in women [58]. As of 2021, HPV positivity has been reported in approximately 33% of HNSCC cases, with nearly 90% of HPV-positive tumours originating in the oropharynx. HPV-associated disease has also been detected, albeit at lower frequencies, in the oral cavity, larynx, nasopharynx, and sinonasal region [58]. Like cervical cancer, HPV16 is the predominant genotype, accounting for the majority of HPV-positive HNSCC and laryngeal tumours, while HPV6, 18, and 45 have been identified in a smaller proportion of cases [59,60,61]. Epidemiological studies demonstrate a rising incidence of oropharyngeal squamous cell carcinoma, particularly involving the tonsils and base of the tongue, among men under 50 years of age with no prior history of tobacco or alcohol use [60]. However, disease outcomes are significantly poorer in individuals who smoke or consume alcohol, highlighting the synergistic impact of traditional risk factors [62]. Oral HPV infection is commonly detected within three months of exposure, following oral or vaginal sexual contact [44]. HNSCC exhibits substantial genetic and molecular heterogeneity, which influences disease behaviour and therapeutic vulnerability [63]. Frequently altered genes include CDKN2A, TP53, PIK3CA, NOTCH1, HRAS, and FBXW7 [64], with approximately 30% of tumours harbouring mutations in regulators of squamous differentiation, such as NOTCH1, IRF6, and TP63 [65]. In addition, HNSCC often demonstrates overexpression of clinically relevant therapeutic targets, including PDL1, CDK6, MET, EGFR, and PGF. Distinct molecular differences are observed between HPV-positive and HPV-negative tumours. HPV-positive HNSCC is characterised by a lower mutational burden, preserved p53 pathway functionality, increased cellular proliferation, and frequent alterations in the PTEN/PIK3CA signalling pathway [65]. These tumours commonly exhibit E2F1 amplification alongside mutations in PTEN, TRAF3, and PIK3CA. In contrast, HPV-negative HNSCC more frequently harbours TP53 mutations, disruption of the G1/S cell-cycle checkpoint, and CDKN2A/B deletion or CCND1 amplification. Unlike HPV-negative tumours, proliferation in HPV-positive cancers is driven by viral oncoproteins, particularly E7, which promotes cell-cycle progression and upregulates cell-cycle regulatory genes [57,66,67].

### 7.3. HPV-Associated Malignancies in HIV-Positive and Immunocompromised Populations

Individuals living with human immunodeficiency virus (HIV) experience a substantially increased burden of HPV-associated malignancies, reflecting impaired immune surveillance, persistent high-risk HPV infection, and reduced viral clearance [68]. Epidemiological studies consistently demonstrate elevated incidence rates of multiple anogenital cancers as well as oral and oropharyngeal squamous cell carcinomas in HIV-positive populations compared with the general population. Among these, anal cancer represents one of the strongest HPV-associated malignancies in HIV-positive individuals, with incidence rates several-fold higher than those observed in HIV-negative populations, particularly among men who have sex with men. HIV–HPV co-infection is also associated with increased prevalence of high-risk HPV genotypes, frequent multi-type infections, and accelerated progression from premalignant lesions to invasive disease [69]. Notably, despite the widespread use of effective antiretroviral therapy (ART), the incidence of HPV-associated cancers, especially anal and oropharyngeal cancers, remains disproportionately high in HIV-positive individuals, suggesting that immune reconstitution does not fully restore HPV-specific immune control [70]. Collectively, these observations highlight the critical role of host immune competence in HPV-mediated carcinogenesis and underscore HIV-positive populations as a key high-risk group for HPV-driven anogenital and oropharyngeal malignancies, in contrast to other cancers where HPV involvement remains inconsistent or controversial.

### 7.4. Cancers with Emerging or Controversial Association with HPV

#### 7.4.1. HPV and Oesophageal Cancer

Oesophageal cancer (EC) is a significant global health burden, with approximately 500,000 new cases and ~406,000 deaths annually [71]. Two main histological subtypes are recognized: oesophageal squamous cell carcinoma (ESCC) and oesophageal adenocarcinoma (EAC). EAC incidence is rising in Western countries, largely associated with Barrett’s oesophagus, while ESCC remains the predominant form worldwide. Risk factors for ESCC include tobacco smoking, alcohol consumption, dietary carcinogens, micronutrient deficiencies, and immune suppression, such as in HIV-infected or transplant patients [72]. The potential role of HPV in ESCC has been investigated using techniques such as PCR, in situ hybridization (ISH), Southern blotting, and serology. However, meta-analyses indicate that these detection methods do not consistently demonstrate a significant association between HPV infection and ESCC [73], and some methods, such as Southern blot, are replaced with contemporary methods. Unlike cervical and oropharyngeal cancers, p16 overexpression does not reliably indicate HPV involvement in oesophageal cancer, suggesting that HPV-driven carcinogenesis is unlikely to play a major role in ESCC. Overall, the evidence linking HPV to oesophageal cancer remains limited, inconsistent, and not sufficient to establish causality. In summary, while HPV DNA can occasionally be detected in ESCC tissues, its etiological role in oesophageal carcinogenesis remains uncertain, and more rigorous epidemiological and mechanistic studies are needed.

#### 7.4.2. HPV and Lung Cancer

Lung cancer contributes to elevated mortality rates in both sexes. In 2012, approximately 1.8 million individuals were impacted by lung cancer, resulting in 491,200 female and 1,098,700 male fatalities, respectively [74]. The primary symptoms of this disease include fatigue, coughing, dyspnea, and hemoptysis. Approximately 40% of lung cancer patients are diagnosed at stage IV, while around 30% are diagnosed at a very advanced stage. Patients diagnosed at an advanced stage exhibited a reduced survival rate, with a five-year survival rate of only 16% [75]. There are two primary types of lung cancer: small-cell lung cancer (SCLC) and non-small-cell lung cancer (NSCLC). The SCLC accounts for roughly 20% of lung infections, while NSCLC constitutes approximately 80% of lung disease. Non-small cell lung cancer (NSCLC) is categorized into three main subtypes: squamous cell carcinoma, adenocarcinoma, and large-cell carcinoma. While small cell and squamous cell carcinomas are strongly associated with smoking, adenocarcinoma is more commonly observed in non-smokers. Beyond tobacco exposure, additional risk factors have been identified, including infectious agents and genetic conditions, with HPV being among the reported contributors [76]. Several studies have investigated HPV as a potential contributor to lung carcinogenesis. HPV DNA, including high-risk types such as HPV16, HPV18, and HPV31, has been detected in lung tumor tissues, bronchial aspirates, peripheral blood, and exhaled breath condensates of some patients. Mechanistic studies suggest that HPV E6 and E7 oncoproteins can inactivate tumour suppressors p53 and pRb, potentially promoting uncontrolled cell proliferation. Some reports note higher E6 expression in adenocarcinoma tissues from females and geographic differences, with higher HPV prevalence in Asia than Europe [77,78,79]. However, it is important to note that the causal role of HPV in lung cancer remains controversial and unconfirmed. While HPV DNA is detectable in a subset of lung cancers, the findings are inconsistent, and no robust epidemiological or mechanistic evidence demonstrates HPV as a definitive etiological factor. Most studies are small, heterogeneous, and observational, and tobacco exposure remains the predominant risk factor for lung carcinogenesis [77]. In conclusion, HPV presence has been observed in lung cancer tissues, but its contribution to lung tumor development should be interpreted with caution until larger, well-controlled studies clarify potential causality.

#### 7.4.3. HPV and Brain Tumour

Glioblastoma multiforme (GBM) is the most common and most aggressive primary malignant brain tumour in adults, classified as a WHO grade IV glioma. It accounts for approximately 50–60% of all malignant neuroepithelial tumours of the central nervous system and is associated with a poor prognosis despite multimodal therapy including surgery, radiotherapy, and chemotherapy. Median overall survival for GBM patients is typically 12–15 months, and the five-year survival rate remains very low (~5–10%) [80]. GBM most often arises in the cerebral hemispheres of adults between ~45–70 years of age, although other brain tumours such as medulloblastoma, ependymoma, and brain stem tumours occur in paediatric populations. Medulloblastoma is typically located in the cerebellum and can metastasize within the central nervous system. Meningiomas, another common variety of brain tumour, arise from the meninges (the protective membranes surrounding the brain and spinal cord). To date, no consistent environmental or lifestyle risk factors (e.g., smoking, alcohol, diet) have been conclusively linked to glioblastoma incidence, and the underlying causes remain largely unknown. Genetic and molecular features such as IDH mutation status, MGMT promoter methylation, and other alterations are important prognostic factors influencing treatment response [80]. There have been observational reports detecting HPV DNA in glioblastoma tissues, and a small number of studies have suggested a possible relationship between HPV detection and prognosis in GBM patients. For example, retrospective analyses have identified HPV genomic sequences within a subset of GBM samples and, in some cases, correlated HPV presence with clinical outcomes [81]. However, the evidence for a causal role of HPV in gliomagenesis remains limited and inconclusive. Unlike high-risk HPV’s role in anogenital and oropharyngeal cancers, current data do not support HPV as a confirmed etiological factor in the development of primary brain tumours. Findings to date are based primarily on small case series and PCR-based detection of viral DNA, which may reflect incidental presence rather than causation. Larger, well-controlled epidemiological, molecular, and mechanistic studies are required to clarify whether HPV contributes to GBM pathogenesis [82].

#### 7.4.4. HPV and Cutaneous Squamous Cell Carcinoma

Cutaneous squamous cell carcinoma (cuSCC) is a common form of non-melanoma skin cancer, with established risk factors including ultraviolet (UV) radiation exposure, immunosuppression, and fair skin phenotype. While HPV is a well-established etiological agent in condyloma acuminata, verruca vulgaris, anogenital cancers, and a subset of head and neck cancers, its role in cuSCC is considered indirect and co-carcinogenic rather than causative. A significantly higher prevalence of HPV has been detected in cuSCC tumours from immunosuppressed individuals, particularly organ transplant recipients, compared with immunocompetent populations [83]. In cutaneous malignancies, HPV is thought to act as a co-factor that enhances susceptibility to UV-induced carcinogenesis, rather than serving as a primary oncogenic driver. Mechanistically, HPV interferes with apoptotic pathways and cellular DNA repair processes, thereby increasing keratinocyte vulnerability to UV-mediated genomic damage. HPV DNA has been detected in sun-protected skin and in clinically normal tissue, supporting the concept of widespread cutaneous HPV colonisation. Viral load analyses indicate that HPV DNA is present in only a small proportion of tumour cells—estimated at 1 in 50 to 1 in 5000 cells—suggesting that HPV is not required for tumour maintenance. Consistent with this, HPV DNA has also been identified in plucked hair follicles from individuals with and without skin cancer, highlighting the ubiquity of cutaneous HPV infection [84]. Molecular studies using degenerate and nested PCR techniques have reported HPV DNA in 32–87% of immunosuppressed individuals and 12–59% of immunocompetent individuals in samples of non-lesional skin [85]. Collectively, these findings support a model in which HPV contributes to early carcinogenic events, particularly in the context of immunosuppression and UV exposure, but is not essential for the persistence or progression of established cuSCC.

#### 7.4.5. Other Cancers

In addition to several cancers, HPV DNA has occasionally been detected in other tumor types, including breast and prostate cancers. Some umbrella and meta-analyses of observational studies report statistically significant associations between HPV presence and breast cancer risk. However, these findings are inconsistent and heterogeneous, and there is currently no evidence to establish a causal relationship. Larger, well-controlled studies are required to clarify any potential role of HPV in breast tumorigenesis [86]. Other systematic reviews indicate the presence of HPV DNA in some prostate cancer specimens. However, the evidence does not reliably support a direct causal role for HPV in prostate carcinogenesis. Observed associations may reflect incidental infection rather than etiological significance [87]. In summary, the presence of HPV in breast and prostate tumors remains an area of ongoing investigation, and any statements regarding causality must be interpreted with caution.

## 8. Prevention for HPV-Associated Cancers

### 8.1. Primary Prevention by Vaccination

HPV not only induces malignancies but also results in many benign diseases, including warts. The World Health Organization has approved many vaccinations that protect against HPV strains 16 and 18. HPV vaccinations have demonstrated effectiveness in decreasing infections at several anatomical areas susceptible to HPV, including the most prevalent cervical cancer, as well as the anal and oral regions. The global administration of the HPV vaccine might reduce the incidence of cervical cancer by as much as 90% [88]. Furthermore, these vaccinations may diminish the necessity for screenings and subsequent medical treatments, including biopsies and invasive procedures linked to unsatisfactory cervical screening outcomes [89]. Vaccination safeguards individuals against HPV types targeted by the vaccine and reduces the frequency of other HPV-related diseases within the community, resulting in fewer illnesses among unvaccinated persons, a process referred to as herd immunity. Research conducted in the United States on females aged 20–29 years revealed that around ten years post-vaccine introduction, the prevalence of HPV types targeted by the vaccination diminished in both vaccinated and unvaccinated women [90]. Cervarix and Gardasil are excellent prophylactic vaccines against HPV infections brought on by some high-risk and low-risk HPV strains 16, 18, 6, and 11 [91]. Table 1 summarizes the classification of HPV types by oncogenic potential, associated clinical manifestations, and current vaccine coverage for different HPV types. HPV preventive vaccines are most efficacious when administered just before viral exposure, in clinical trials, in young adults, and precocious adolescents. The vaccines provide near 100% protection against adenocarcinoma in situ (AIS), cervical intraepithelial neoplasia (CIN), and genital warts with near 100% efficacy in HPV negative populations [92].

Table 2 Summarizes selected HPV vaccine clinical trials. Vaccines provide good protection but slightly reduced efficacy even in subjects with previous HPV exposure.

### 8.2. Effectiveness and Recommendations for Different Age Groups

#### 8.2.1. Adolescents and Pre-Adolescents (9–14 Years) Effectiveness

HPV vaccines work best if given before exposure to the virus. In this age group, vaccination produces a robust immune response, and the vaccine protects against the most common HPV types producing cervical cancer, other cancers, and genitourinary warts (anal, penile, oral, vulvar cancers) [91]. The vaccine prevents infection with high-risk strains of HPV 90–100% when given before exposure [99].

#### 8.2.2. Young Adults (15 to 26 Years) Effectiveness

This age group is more protected compared to young people, though the protection is slightly reduced in case the individual has been exposed to one or more HPV strains before vaccination [91]. It could still protect against some HPV types that the person is not exposed to. In this age group, people should complete the vaccine series (two doses for 15–26 years old and three doses if started after age 15) [99]. Booster vaccination is recommended for all people up to age 26 who were not vaccinated or who haven’t completed the vaccination course.

#### 8.2.3. Adults (Ages 27–45) Effectiveness

For adults in this age group, the vaccine might still provide a little protection, although the advantage is diminished as adults are less likely to be HPV-naive (i.e., they might have been exposed to some HPV types already). The vaccine might still prevent infection with non-infected HPV types, therefore lowering the risk of cancer along with other HPV-related diseases [91]. The Center for Disease Control and Prevention (CDC) suggests adults 27–45 should consult their healthcare provider about the vaccine. This is especially important among people at increased risk (for example, partners with many sexual partners or weakened immune systems). Adults over 26 aren’t usually recommended to get the vaccine, though it may be helpful for some people.

#### 8.2.4. Senior Adults (Ages 46+) Effectiveness

In adults more than forty years old, the vaccine isn’t recommended as part of regular public health guidelines as it’s unlikely to provide lots of benefit [91]. This is because people of this age group are more likely to have had at least one HPV strain injected already. The vaccine is not usually advised for adults over the age of 46 years unless they are at special risk (such as immunocompromised patients), in which case vaccination should be considered on an individual basis by a healthcare provider.

### 8.3. Secondary Prevention by Diagnostics and Screening

For the most predominant cancer type, secondary prevention relies on population-based cervical cancer screening, which enables the detection of precancerous lesions and early-stage disease. Routine screening includes Papanicolaou (Pap) testing using liquid-based cytology, HPV DNA testing, and other adjunctive diagnostic approaches, as summarised in Table 3 [100]. Screening strategies and recommendations vary across countries; therefore, adherence to national and regional guidelines is essential. Current recommendations generally advise that individuals aged 25–65 years undergo primary HPV testing every five years. In settings where primary HPV testing is not widely available, co-testing (HPV testing plus cytology) every five years or cytology alone every three years remains an acceptable alternative [101]. The initiation of screening at 25 years reflects evolving evidence supporting the use of high-sensitivity HPV-based assays and adjustments in screening intervals. Women living with HIV are at a significantly increased risk of persistent HPV infection and cervical cancer; consequently, screening in this population may commence at an earlier age and occur at shorter intervals to enable timely detection and management [102].

**Table 2:** Ongoing and completed clinical trials of HPV Vaccines.

Study Number	Vaccine Tested	Phase	Sponsor	Inclusion Criteria	Study Type & Participant Count (PC)	Main Outcome	Reference
NCT04180215	HB-200 (arenavirus-based)	I/II	HOOKIPA Pharma	HPV16+ recurrent/metastatic head and neck cancer; prior treatment with pembrolizumab	Open-label, multicentre PC—35 (20 first-line, 15 second line)	Safety, tolerability, and preliminary efficacy of HB-200 in combination with pembrolizumab	[103]
NCT03418480	BNT113 (HARE-40)	I/II	University of Southampton & BioNTech SE	HPV16+ head and neck, cervical, or anogenital cancers; prior treatment with chemotherapy or immunotherapy	Dose escalation, open-label PC—Not specified	Safety, tolerability, and recommended dose of BNT113	[104]
NCT03180034	Cervarix (bivalent)	Not specified	National Cancer Institute (NCI)	Girls aged 9–14 years; no prior HPV vaccination	Immunobridging PC—1240 (620 girls, 620 women)	Immunogenicity of a single dose of HPV vaccine compared to three doses of Gardasil	[105]
NCT06623409	V540B (next-gen HPV vaccine)	I	Merck & Co., Inc.	Healthy adults aged 18–45 years; no prior HPV vaccination	Open-label, multicentre PC—Not specified	Safety and tolerability of V540B in healthy adults	[106]
NCT03180684	VGX-3100 (DNA vaccine)	II	Inovio Pharmaceuticals	Women with vulvar HSIL (VIN 2 or VIN 3) associated with HPV types 16 and/or 18	Randomized, open-label PC—Not specified	Efficacy of VGX-3100 in preventing progression to vulvar cancer	[107]
NCT04965350	Bivalent HPV vaccine (Types 16, 18)	III	Shanghai Zerun Biotechnology Co., Ltd.	Healthy females aged 9–30 years	Randomized, double-blind PC—not specified	Immunogenicity and safety of three consecutive lots of bivalent HPV vaccine	[108]

**Table 3:** Screening types for HPV infection.

Type Screening/Diagnosis	Description	Indication
Next-Generation Sequencing (NGS)	Thorough examination of the HPV genome alongside host genetic variations.	Investigating new biomarkers and comprehending the integration of HPV.
HPV DNA Testing	Identifies the existence of high-risk HPV DNA within cervical cells.	Evaluation for females between the ages of 30 and 65, particularly for those presenting with atypical Pap smear results.
E6/E7 Oncoprotein Detection	Identifies E6 and E7 oncoproteins from high-risk HPV types, signifying active viral oncogenesis. Examination of circulating tumour DNA (ctDNA) or alternative markers present in blood or various body fluids.	Evaluation of HPV-positive women, determining the likelihood of disease advancement. Clear evidence of cancer-causing potential, with a strong focus on specificity.
Pap Smear (Papanicolaou Test)	Gathering cellular samples from the cervix and conducting microscopic analysis to detect any irregularities.	Regular screening for women between the ages of 21 and 65, particularly for those who are sexually active
Digital Cervicography	A method involving the capture and examination of cervical images to identify any irregularities.	Assessment of atypical cytology or HPV test outcomes, subsequent evaluation for VIA/VILI findings

#### 8.3.1. Cervical Sampling

The self-sampling method for cervical cancer screening has significant potential to enhance screening coverage [109]. The availability of self-collection of samples may incentivize more women to participate in cervical screenings, particularly in low- and middle-income countries where taboos and accessibility challenges hinder women’s access to healthcare facilities [110]. A comprehensive evaluation was conducted on data from studies concerning HPV self-sampling equipment, with publications published from 2013 to October 2023, retrieved from multiple databases. Out of the 70 publications, 22 addressed diagnostic accuracy, 32 focused on acceptability, and 16 covered both topics. The most prevalent devices included the Evalyn Brush, FLOQ Swab, Cervex-Brush, and Delphi Screener. Of the 38 investigations on diagnostic accuracy, 94.7% demonstrated that self-collected specimens had equal sensitivity and specificity to those obtained by clinicians. The acceptability of the gadgets varied between 84.2% and 100%. They determined that self-sampling had significant potential to enhance cervical cancer screening participation [109]. HPV testing may be conducted on a self-collected vaginal sample, hence improving screening coverage [111].

#### 8.3.2. Anal Screening

Kimura et al. evaluated multiple screening strategies for the detection of high-grade squamous intraepithelial lesions (HSIL), using high-resolution anoscopy (HRA) as the reference standard. Anal cytology alone demonstrated a sensitivity of 73%, which increased to 85% when combined with HPV testing; however, specificity declined from 73% to 43% with the addition of HPV testing. Based on these findings, the authors proposed an optimal screening algorithm consisting of initial anal cytology, followed by HPV testing as a triage tool for individuals with normal cytology or atypical squamous cells of undetermined significance (ASC-US). Patients with oncogenic HPV detected by this approach were subsequently referred for HRA. This algorithm achieved an overall sensitivity of 80% and specificity of 71% [112]. In addition to clinician-based screening, anal HPV self-sampling and digital anorectal examination (DARE) have emerged as complementary strategies for the early detection of anorectal malignancies, particularly in high-risk populations [113].

#### 8.3.3. Human Papillomavirus DNA Testing

HPV testing is an essential component of screening and may be conducted utilizing DNA/RNA PCR-based methodologies, which exhibit high sensitivity and specificity in identifying HPV DNA or RNA [114]. Upon detecting malignancy in Pap smear findings, concurrent HPV DNA testing on the same cytology sample is performed due to its superior sensitivity and specificity in diagnosis [115]. Cervical cancer associated with HPV arises from pre-cancerous lesions, but non-HPV-related cervical cancer seldom has detectable precancerous lesions. In accordance with WHO standards, an HPV DNA test is conducted to detect HPV-associated cervical cancer. To discriminate between the two morphological kinds, it is recommended to utilize p16 IHC, as morphology alone is insufficient for their distinction [116].

## 9. Therapeutic Approaches for HPV-Associated Cancers

### 9.1. Surgery

Surgery is used frequently for HPV-associated cancers, especially if the cancer is localized (early stage). Surgical options may involve the removal of tumours, local tissue, and lymph nodes with the goal of total cancerous cell removal. Excisional surgery is one type in which the removal of the tumour with a healthy tissue margin to reduce recurrence risk. Another type is laparoscopic surgery, which is a minimally invasive approach through small incisions and a camera, often utilized in the workup of HPV-associated cancers (e.g., cervical cancer). Radical surgery, which is more extensive for big or advanced tumours, for example, in advanced cervical cancer radial hysterectomy might be indicated. Laser surgery, which is mostly used for cancerous lesions of the cervix and vulva, where lasers can excise abnormal cells. The last surgical option is lymph node dissection, in which nearby lymph nodes are removed for staging & to see if cancer has spread [117].

### 9.2. Radiation Therapy

Radiation therapy kills cancer cells or shrinks tumours with high-energy rays or particles. It is especially useful in the treatment of HPV-related cancers, whether localized or not invasive to distant organs. Radiation could be used as primary therapy or following surgery to kill any remaining cancer cells. The most common form is external beam radiation, in which radiation comes from outside the body and hits the tumour directly. Internal radiation (Brachytherapy) is used in some cervical cancer, in which radioactive sources are positioned near or within the tumour. Palliative radiation is useful for pain management and shrinking tumours in advanced-stage cancers [118].

### 9.3. Chemotherapy

The use of certain drugs to kill or inhibit cancer growth is called chemotherapy. It is typically used if the cancer is advanced beyond the primary site or if radiation or surgery are not adequate in themselves. It is used in the treatment of advanced HPV-associated cancers, such as cervical cancer, if the disease has reached other organs. Chemotherapy has a potential role in all stages of cancer. Chemotherapy can be administered in the neoadjuvant, concurrent with radiation, adjuvant, and palliative settings. Randomised evidence has clearly established the role of chemotherapy for uterine cervical cancer in conjunction with radiation and metastatic/palliative settings. Additionally, it is also used to increase the tumour susceptibility to radiation therapy. Metastatic cancers are also treated with chemotherapeutic drugs. Chemotherapy drugs like cisplatin and carboplatin are useful in treating cervical cancer. HPV cancers, including head and neck cancers, are usually treated with paclitaxel and 5-fluorouracil 5-FU [119].

### 9.4. Targeted Therapy

Drugs targeting cancer cells without affecting normal cells are referred to as targeted therapies. These therapies may target molecules that control the growth and survival of cancer cells. Targeted therapies are not yet common in HPV-associated cancers, although in cases where traditional therapies (surgery/radiation/chemo) fail, they are increasingly used. For instance, the drug Bevacizumab, which inhibits blood supply to tumours, is sometimes used with chemotherapy in treating cervical cancer [120].

### 9.5. Immunotherapy

Immunotherapy is a promising strategy to stimulate or model the body’s disease-fighting capability to combat cancer [121]. It shows some success for cancers of viral etiology, such as HPV-associated cancers. Checkpoint inhibitors include drugs that inhibit PD-1 and are approved for the treatment of some HPV-related cancers, including oropharyngeal tumours and resistant cervical cancer [122]. They are used for advanced or recurrent cancers only, and in case of failure of traditional therapies.

### 9.6. New and Improved Treatments for HPV-Related Cancers

Significant advancements have been made in the treatment of HPV-driven cancers in recent years (2024 and 2025) primarily because of immunotherapies, therapeutic vaccinations, and innovative combination regimens.

### 9.7. Immunotherapy and Combination Regimens

#### 9.7.1. Immune Check-Point Inhibitors-Based Immunotherapy

Immune checkpoint blockade (ICB) has become an integral part of the treatment of cervical cancer. Pembrolizumab (anti–PD-1) in combination with chemoradiotherapy (CRT) has been approved by the FDA recently [123] based on the Phase 3 KEYNOTE-A18 trial done by [124], which proved that using standard CRT along with pembrolizumab improved progression-free survival (PFS)—67.8% vs. 57.3% PFS at 24 months (HR = 0.70, *p* < 0.01). Immunotherapy based on PD-1 inhibitor, cemiplimab, demonstrated a higher survival rate as compared to chemotherapy in 2nd line metastatic cervical cancer [125]. HNSCC, particularly oropharyngeal cancer, demonstrated a positive response to vaccine-based immunotherapy along with chemotherapy. Examination of PDS0101 along with pembrolizumab in first-line metastatic HPV16-positive HNSCC is in phase 2 of the VERSATILE-002 trial, which showed enhanced anti-tumour activity as compared to pembrolizumab alone and was significantly non-toxic at the same time. The success of which resulted in phase three of the VERSATILE-003 trial, to compare vaccine + PD-1 vs. PD-1 alone [126]. In parallel, BNT113: an mRNA vaccine targeting HPV16 oncoproteins developed by BioNTech, is being evaluated with pembrolizumab in the AHEAD-MERIT trial for first-line HPV+ HNSCC [127]. In 2025, a triplet immunotherapy trial: PDS0101 vaccine along with tumour-targeting interleukin 12 antibody-drug conjugate and bintrafusp alfa (a bifunctional anti–PD-L1/TGF-β transforming growth factor was carried out and improved ORR in patients associated with HPV-16 cancers [126]. With checkpoint inhibitors, therapeutic vaccines, and novel combination regimens showing notable improvements in clinical outcomes, these developments collectively highlight the critical importance of immunotherapy in the treatment of HPV-associated cancers. Immunotherapy’s incorporation into routine treatment regimens holds significant potential for improving survival and quality of life for patients with HPV-driven malignancies as current trials continue to hone these approaches. Table 4 summarizes pivotal clinical trials evaluating checkpoint inhibitors, therapeutic vaccines, and combination immunotherapy regimens targeting HPV-driven malignancies.

#### 9.7.2. New Therapeutic Vaccine and Immunotherapy

Novel therapeutic vaccines are being paired with immunotherapy and conventional treatment to boost the efficacy of combination therapies. A liposomal therapeutic vaccine: PDS0101 (Versamune HPV vaccine) against HPV16 E6/E7 antigens is in phase 2 IMMUNOCERV trial along with chemoradiation, produced remarkable 3-year outcomes in locally advanced cervical cancer patients; a 36-month overall survival of 84.4% (100% in patients who received all vaccine doses), compared to ~64% historically with chemoradiation alone [133]. In a recurrent or metastatic cervical cancer objective response rate (ORR) of 53.8% was reported by a trial of Sintilimab (PD-1 inhibitor) in combination with quadrivalent Gardasil (a prophylactic HPV16/18 cancer vaccine) which is par then immunotherapy alone with very less toxicities: ≥Grade 3 and median follow up duration was 16.07 months (range: 3.64–48.2 months) [134].

#### 9.7.3. Antibody–Drug Conjugates and Immunotherapy

For recurrent cervical cancer, targeted therapies are expanding options. Tisotumab vedotin, an antibody drug conjugate (ADC) which targets the tissue factor, showed improved overall survival rate in the phase three innovaTV301 trial as compared to chemotherapy [124] and confirmed the greater survival chances in recurrent cervical cancer. trastuzumab deruxtecan (T-DXd), a HER2-directed ADC, which is a tumour-specific molecular target, showed the 50% ORR in a cohort study of 40 cervical cancer patients with HER2 expression (IHC 2+/3+). Conspicuously, the ORR was 75% in patients with high HER2 expression (IHC 3+) [124]. According to the findings of the DESTINY-PanTumor02 trial, which was published in 2023–2024, T-DXd may be used as a tumour-agnostic treatment for HER2-positive tumours, including some types of cervical cancer. Their efficacy in cervical cancer represents a breakthrough for patients with refractory illness, as these ADCs provide a targeted administration of a payload similar to chemotherapy [135].

### 9.8. Adoptive Cell Therapies

**Figure 6:**
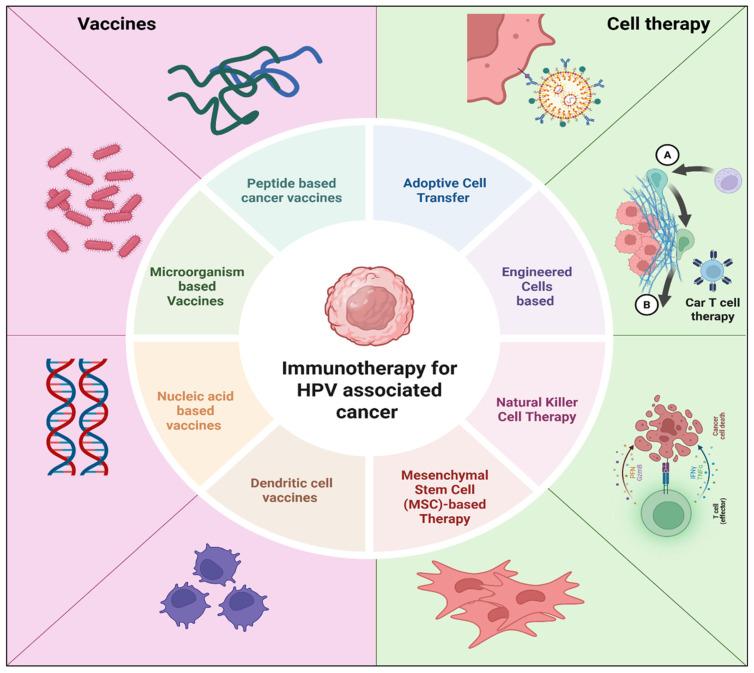
An illustration of immunotherapeutic strategies for HPV-associated cancers.

The first FDA approval of a tumour-infiltrating lymphocyte (TIL) therapy (Lifileucel) for solid tumours such as melanoma was also announced in 2024, which opened the door for similar approaches in cancers linked to HPV [136]. Tumour-infiltrating T cells from a patient are extracted, grown ex vivo, and then reinfused as part of TIL treatment. Preliminary research on HPV targeted TIL for cervical cancer has revealed significant responses in a subgroup of patients. For example, in a trial with TILs chosen for HPV16/18 reactivity in metastatic cervical cancer, two patients saw complete, long-lasting tumour regressions, and approximately 28% of patients who had received extensive pretreatment showed an objective response [136]. These results are encouraging, even if most people only have minor toxicity, which primarily stems from the lymphodepleting regimen. To assess lifileucel TIL treatment in conjunction with pembrolizumab for frontline cervical cancer, a global phase 3 trial (TILVANCE-301) was started. Adoptive T-cell treatments may soon be feasible choices for HPV-driven malignancies [136]. Figure 6 summarizes the key immunotherapy modalities under investigation for HPV-driven malignancies, categorized into vaccine-based and cell-based approaches. Vaccine-based strategies include peptide-based cancer vaccines, microorganism-based vaccines, nucleic acid-based vaccines, and dendritic cell vaccines, aimed at inducing HPV-specific immune responses. Cell-based therapies encompass adoptive cell transfer (ACT), engineered T cell therapies (CAR-T/TCR-T), natural killer (NK) cell therapy, and mesenchymal stem cell (MSC)-based therapy. These strategies are designed to overcome immune evasion mechanisms and enhance tumour-specific cytotoxicity, thereby improving clinical outcomes in HPV-related cervical, oropharyngeal, and other epithelial cancers.

### 9.9. Other Systemic Therapies and Combination Therapies

Engineered T-cell treatments (TCR-T cells) are being tested against HPV16 E7 in HNSCC in several early-phase trials [137]. It is important to remember that conventional treatments for HPV-associated head and neck cancers, such as surgery, radiation, and platinum chemotherapy, are still effective. Rather than developing completely new drug classes, better results are probably going to be achieved by combining the vaccines and immunotherapies together to achieve enhanced patient outcomes. Currently, surgery combined with chemotherapy and radiation therapy is the major treatment choice for these tumours at the primary stage [138]. The World Health Organisation (WHO) has set the monumental target of vaccinating 90% of young girls to eradicate cervical cancer worldwide by 2030. Immunotherapy can further be explored to enhance the results in recurrent or metastatic stages. Optimising vaccine programs, screening, and therapies is necessary to reach this aim, especially in lower-middle-income countries, where low screening and early detection rates have made cervical cancer a significant cause of cancer-related death among women [139].

## 10. Advances in Disease Screening Methods

### 10.1. Enhanced Molecular Evaluation

To determine which women truly require treatment, better evaluation approaches are required, as HPV testing finds more women at risk. The discovery of molecular biomarkers has been prompted by the low sensitivity and reproducibility of cytology (Pap smear), which has historically been used to triage women with HPV [140]. The use of DNA methylation markers to anticipate the occurrence of high-grade cervical lesions is one significant advancement. A test called WID-qCIN, which measures the methylation of host genes (including DPP6, RALYL, and GSX1) in HPV-positive samples, was assessed in a sizable real-world study published in Nature Medicine in 2024 [141]. Furthermore, p16/Ki-67 dual-stain cytology, which identifies transforming HPV infections, has demonstrated better triage accuracy than Pap cytology alone, improving specificity and risk stratification among HPV-positive women. These markers are increasingly integrated into screening algorithms as evidence for their clinical utility grows [142].

### 10.2. Early Detection of Biomarkers

The significance of circulating HPV DNA as a biomarker for early detection and surveillance in HPV-associated oropharyngeal malignancies has been established by recent studies. Months before clinical symptoms manifest, increasing HPV DNA levels in plasma following therapy may be a sign of recurrence [143]. Combining saliva and blood tests improves detection sensitivity; one study found that early-stage HPV-positive tumours may be identified with 100% sensitivity. Oral rinse HPV DNA tests are being researched for early cancer detection in high-risk patients, but not commonly. Salivary genomic tests, which target DNA mutations or methylation, have demonstrated encouraging results in early identification of HPV-negative oral malignancies. With 2024 studies confirming its predictive significance and role in therapy de-intensification techniques, p16 immunohistochemistry is a standard surrogate marker for HPV-related HNSCC in clinical pathology [144].

### 10.3. Next-Generation Sequencing

The q-PCR technique, frequently utilized for the detection of HPV DNA in biological samples, cannot accurately pinpoint specific molecular markers. Conversely, Next-Generation Sequencing (NGS) represents a powerful method that provides an extensive analysis of HPV DNA in tumours [145]. This involves ascertaining the complete viral genome sequence, alterations in the host’s chromosomes (including amplifications and deletions), integration frequency, specific sites of integration within chromosomes, and the identification of adjacent genes [145]. Next-generation sequencing methods enable accurate identification of viral sequences associated with tumours, including specific strains of HPV16 or other genotypes, which serve as important tumour markers [146].

### 10.4. Technology and AI in Screening

Traditional screening methods for HPV have been strengthened by technological advancements. For communities with limited resources, HPV testing is being paired with point-of-care systems (such as smartphone-based visualisation and quick HPV assays). Artificial intelligence (AI) is also being used in cervical screening; for instance, machine learning algorithms can identify precancers by analysing digital cytology slides or cervix photos. AI-assisted screening can meet or surpass experienced readers in accuracy, according to meta-analyses conducted in 2023 and 2024 [147]. In 2024, CerviSCAN, an AI system, demonstrated sensitivity comparable to that of cytotechnologists in automatically identifying high-grade lesions on Pap test images. Standardising the interpretation of cervical lesions was made possible by CerviCARE, another AI technology for automated colposcopy picture analysis [148]. These techniques, which are still being tested, have the potential to decrease human error and extend expert-level screening to regions with few specialists [149]. In conclusion, the screening paradigm is shifting towards more tech-enabled, self-driven, and molecular approaches, which will improve early illness diagnosis caused by HPV.

## 11. Conclusions and Future Prospects

Human papillomavirus represents a critical public health challenge due to its etiological role in a range of epithelial malignancies, most notably cervical, oropharyngeal, and anogenital cancers. The oncogenicity of high-risk HPV types, primarily mediated by the viral oncoproteins E6 and E7 and the subsequent integration of viral DNA into the host genome, makes the virus capable of subverting host cellular regulation and promoting malignant transformation. Substantial progress has been made in prevention, particularly through prophylactic vaccination and population-level screening programs. Nevertheless, disparities in vaccine access, diagnostic capabilities, and therapeutic resources persist, particularly in low- and middle-income countries, where the burden of HPV-related cancers remains disproportionately high. This review consolidates existing evidence and emerging research on the virology, pathogenesis, and clinical management of HPV-associated cancers, while also highlighting novel diagnostic and therapeutic innovations currently under investigation. Continued investment in public health infrastructure, equitable vaccine distribution, and translational research is imperative to achieve the global objective of HPV-related cancer elimination. As the field advances, an interdisciplinary approach integrating molecular oncology, immunotherapy, and epidemiology will be essential to reducing incidence, improving patient outcomes, and ultimately mitigating the global cancer burden attributable to HPV.

## Figures and Tables

**Table 1:** Classification of HPV types, associated diseases, and vaccine coverage.

HPV Types	Risk Classification	Associated Diseases	Vaccine Coverage	Reference
6, 11	Low risk	Genital warts, laryngeal papillomatosis, oral papillomas	Quadrivalent (Gardasil), Nonavalent (Gardasil 9)	[10]
16, 18	High risk	Cervical, anal, vulvar, vaginal, penile, and oropharyngeal cancers	Bivalent (Cervarix), Quadrivalent, Nonavalent	[93,94]
31, 33, 45, 52, 58	High risk	Cervical and other anogenital cancers	Nonavalent (Gardasil 9)	[58]
35, 39, 51, 56, 59, 66, 68	High risk	Cervical and other anogenital cancers	Not covered by current vaccines. Next-gen multivalent and L2-based vaccines under research for broader coverage	[95]
26, 53, 73, 82	Probable high risk	Potential association with cervical and other anogenital cancers	Not covered by current vaccines. Broad-spectrum L2-based, DNA, and peptide vaccines under investigation	[96,97]
1, 2, 4, 7, 10, 28, 40, 42, 43, 44, 54, 61, 70, 72, 81, CP6108	Low risk	Common warts, plantar warts, flat warts, and other benign lesions	Not covered by current vaccines. Therapeutic vaccines, live attenuated, and bacterial vector vaccines under investigation	[93,98]

**Table 4:** Ongoing clinical trials of immunotherapy in HPV-associated cancers.

Trial Name	Target	Therapy	Phase	Status/Outcome	Reference
KEYNOTE-A18	Cervical cancer	Pembrolizumab + Chemoradiotherapy (CRT)	Phase 3	FDA-approved, improved PFS and OS	[128]
KEYNOTE-826	Advanced cervical cancer	Pembrolizumab + Chemotherapy + Bevacizumab	Phase 3	FDA-approved, significant OS benefit	[129]
NCT03189719 (ADXS-HPV)	Recurrent/metastatic cervical cancer	Listeria monocytogenes-based immunotherapy (Axalimogene filolisbac)	Phase 3 (Terminated)	Terminated early, informative for live-vector vaccines	[130]
VERSATILE-002/VERSATILE-003	HPV16+ Head and Neck Squamous Cell Carcinoma (HNSCC)	PDS0101 + Pembrolizumab (Therapeutic vaccine + PD-1 inhibitor)	Phase 2/Phase 3	Positive Phase 2 data, Phase 3 ongoing	[126]
AHEAD-MERIT Trial (BNT113)	HPV16+ HNSCC	BNT113 mRNA vaccine + Pembrolizumab	Phase 2	Recruiting, early promising data	[131]
NCT04287868 (Triplet Therapy)	HPV16-associated cancers	PDS0101 + IL-12 Antibody-Drug Conjugate + Bintrafusp alfa	Phase 1/2	Promising ORR, ongoing trials	[132]

## Data Availability

Data sharing is not applicable to this article as no new data were created or analyzed in this study.
